# Cerebrospinal fluid pulse pressure amplitude during lumbar infusion in idiopathic normal pressure hydrocephalus can predict response to shunting

**DOI:** 10.1186/1743-8454-7-5

**Published:** 2010-02-12

**Authors:** Per K Eide, Are Brean

**Affiliations:** 1 Department of Neurosurgery, Rikshospitalet University Hospital, N-0027 Oslo, Norway; 2 Department of Neurology, Vestfold County Hospital, N-3112 Tuensberg, Norway

## Abstract

**Background:**

We have previously seen that idiopathic normal pressure hydrocephalus (iNPH) patients having elevated intracranial pressure (ICP) pulse amplitude consistently respond to shunt surgery. In this study we explored how the cerebrospinal fluid pressure (CSFP) pulse amplitude determined during lumbar infusion testing, correlates with ICP pulse amplitude determined during over-night ICP monitoring and with response to shunt surgery. Our goal was to establish a more reliable screening procedure for selecting iNPH patients for shunt surgery using lumbar intrathecal infusion.

**Methods:**

The study population consisted of all iNPH patients undergoing both diagnostic lumbar infusion testing and continuous over-night ICP monitoring during the period 2002-2007. The severity of iNPH was assessed using our NPH grading scale before surgery and 12 months after shunting. The CSFP pulse was characterized from the amplitude of single pressure waves.

**Results:**

Totally 62 iNPH patients were included, 45 of them underwent shunt surgery, in whom 78% were shunt responders. Among the 45 shunted patients, resistance to CSF outflow (R_out_) was elevated (≥ 12 mmHg/ml/min) in 44. The ICP pulse amplitude recorded over-night was elevated (i.e. mean ICP wave amplitude ≥ 4 mmHg) in 68% of patients; 92% of these were shunt responders. In those with elevated overnight ICP pulse amplitude, we found also elevated CSFP pulse amplitude recorded during lumbar infusion testing, both during the opening phase following lumbar puncture and during a standardized period of lumbar infusion (15 ml Ringer over 10 min). The clinical response to shunting after 1 year strongly associated with the over-night ICP pulse amplitude, and also with the pulsatile CSFP during the period of lumbar infusion. Elevated CSFP pulse amplitude during lumbar infusion thus predicted shunt response with sensitivity of 88 and specificity of 60 (positive and negative predictive values of 89 and 60, respectively).

**Conclusions:**

In iNPH patients, shunt response can be anticipated in 9/10 patients with elevated overnight ICP pulse amplitude, while in only 1/10 with low ICP pulse amplitude. Additionally, the CSFP pulse amplitude during lumbar infusion testing was elevated in patients with elevated over-night ICP pulse amplitude. In particular, measurement of CSFP pulse amplitude during a standardized infusion of 15 ml Ringer over 10 min was useful in predicting response to shunt surgery and can be used as a screening procedure for selection of iNPH patients for shunting.

## Background

The clinical condition normal pressure hydrocephalus (NPH) incorporates gait disturbance, mental deterioration and urinary incontinence, combined with enlarged cerebral ventricles and a normal lumbar cerebrospinal fluid pressure (CSFP) [1]. Usually no cause is identified, in which case the condition is denoted idiopathic NPH (iNPH). Although the pathophysiology of iNPH is disputed [2], previous studies have shown that shunt surgery can be effective, and that clinical improvement can be sustained for years [3-5].

Hydrodynamic tests, in particular the lumbar infusion test, have been used for selecting patients for surgery, although the literature is very divergent concerning its role in iNPH [6]. During lumbar infusion testing, the static CSFP can either be monitored during constant flow infusion, constant pressure infusion or during bolus infusion to the thecal sac. In our practice, we have for many years used a modification of the constant rate infusion test originally described by Katzman [7] for determination of resistance to CSF outflow (R_out_). However, the utility of R_out _in selecting iNPH patients for surgery is controversial [8-13]. On the other hand, we have found that the ICP pulse (that is the amplitude of the single cardiac-beat induced ICP waves) during over-night ICP monitoring is very useful for predicting shunt response in iNPH [14]. Thus, in our previous series of 130 shunted iNPH patients, shunt response was seen in 9 of 10 patients with elevated ICP wave amplitudes but only in 1 of 10 with low ICP wave amplitudes [15].

With regard to lumbar infusion testing, the various approaches (e.g. constant flow, constant pressure or bolus infusion methods) consistently assess the static and not the pulsatile CSFP. Others [16] and our group [8,11,17] have reported experiences from assessing the pulsatile CSFP during lumbar infusion testing. Based on these experiences, it could be anticipated that determining the CSFP pulse during lumbar infusion testing might better characterize the pressure-volume reserve capacity than the static CSFP. Moreover, successful assessment of the pulsatile CSFP during lumbar puncture might represent an advantage, given that lumbar puncture is a low-risk procedure, and more useful in a clinical neurological setting than continuous ICP monitoring. The pulsatile CSFP can be measured during the opening phase of lumbar puncture, as well as during lumbar infusion. Thus, our goal with the present study was to establish a more reliable screening procedure for selection of iNPH patients for shunt surgery, based on determining the CSFP pulse amplitude during lumbar infusion testing. For this purpose, in the present study we explored how measurement of the pulsatile CSFP during lumbar infusion testing correlated with the ICP pulse monitored over-night and with the response to shunting. To do this we retrieved all lumbar infusion tests done during diagnostic work-up for iNPH in this department during the time period 2002-2007. These infusion tests were stored as continuous CSFP raw data (originally sampled at 100-200 Hz). In the present study, these raw data files were re-analyzed; the CSFP pulse amplitude was determined during the opening phase after lumbar puncture and also during a period of lumbar infusion (standardized as 15 ml infusion over 10 min). All patients had their ICP monitored over-night; therefore the infusion test results could be related to the pulsatile ICP recorded over-night, and with the clinical response to shunting.

## Methods

### Patient material

The patient material consisted of all patients being assessed for iNPH at the Department of Neurosurgery, Rikshospitalet University Hospital, during the 6-years period 2002-2007, in whom both over-night ICP monitoring and lumbar infusion testing had been done during the diagnostic pre-operative work-up. The patients were referred from local neurological departments based on their symptoms of gait disturbance, incontinence, and dementia, combined with radiological ventriculomegaly.

For diagnostic work-up the patients were hospitalized for 3 days. Following clinical and radiological assessment on day 1 (day of admittance), ICP monitoring was done from day 2 to day 3. The lumbar infusion test was done on day 3. After discharge from the department on day 3, they returned 1-3 weeks later for surgical treatment provided this was advocated.

This study was approved by the hospital authority of Rikshospitalet University Hospital and by the Norwegian Social Science Data Services. The Regional Committee for Research Ethics was informed in writing, and had no objections to the study.

### Clinical and radiological assessment

Our diagnostic work-up for iNPH patients has previously been described [14,15]. In short, based on findings at neurological examination, the severity of clinical iNPH was graded using our NPH grading scale (scores ranging from 3-15), which assesses the combined severity of gait disturbance, urinary incontinence and dementia. The size of the ventricles was assessed using the linear measure Evan's index [14].

### Diagnostic ICP monitoring and lumbar infusion testing

Diagnostic continuous ICP monitoring was done through a frontal burr hole prepared under local anesthesia. A Codman ICP MicroSensor (Codman, Johnson & Johnson, Raynham, MA, USA) was placed 1-2 cm into the brain parenchyma. The ICP monitoring was done from the evening of day 2 until the morning of day 3. For each patient we used the over-night ICP recording from 11 p.m. to 7 a.m., when the patient was supine in bed.

**Figure 1:**
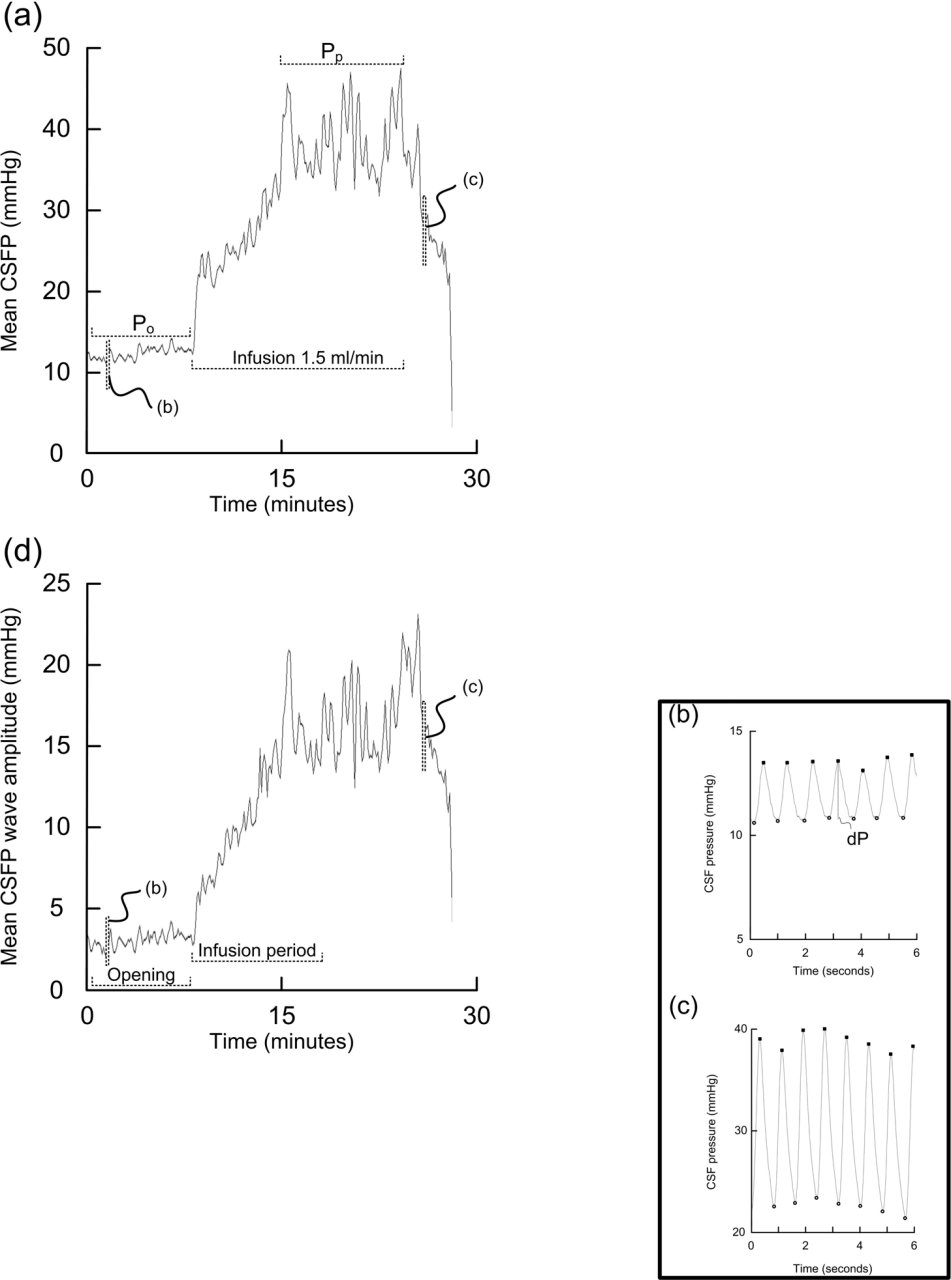
**Analysis of lumbar infusion tests: (a) a plot showing our conventional method of performing a lumbar infusion test, using constant-rate infusion at 1.5 ml/min of Ringer solution**. The plot of mean CSFP against time incorporates the period before infusion (opening pressure; P_o_), and during the infusion period (infusion rate of 1.5 ml/min). The plateau pressure (P_p_) is indicated. The resistance to CSF outflow (Rout) is calculated as (P_p _- P_o_)/infusion rate. Two 6 s time windows of the continuous CSFP signal from (b) the start and (c) the termination of the infusion test are indicated, illustrating the single CSFP pulse waves. (d) Plot of mean CSFP pulse amplitude against time, indicating the period after lumbar puncture and before infusion (opening phase), and the 10 min period during the infusion. For comparison between patients we compared the CSFP pulse amplitude during the first 10 min of infusion.

On the morning day 3, the lumbar infusion test was done, as previously described [11]. Our strategy represents a modification of the original Katzman procedure [7]. The test was performed with the patient in the supine position by making a midline lumbar puncture with a 19-gauge needle between the L3 and L4 vertebrae (one puncture only). The lumbar cerebrospinal fluid (CSF) pressure was measured continuously using the Truwave PX-600F Pressure Monitoring Set (Edwards Life sciences LLC, Irvine, CA, USA), during the opening phase after lumbar puncture (P_o_), and during infusion with a Ringer solution at a standard infusion rate of 1.5 ml/min (fig 1a). The resistance to CSF outflow (R_out_) was calculated as the difference between the plateau pressure (P_p_) and the opening pressure (P_o_), divided by infusion rate [11,18].

### Surgical treatment

The criteria for surgical treatment during this time period were based on a combination of clinical and radiological observations, and ICP monitoring, as previously described [15]. The infusion test was considered abnormal when resistance to CSF outflow (R_out_) was ≥ 12 mmHg/ml/min. Surgical treatment was implantation of a ventriculo-peritoneal (VP) shunt: 46 patients received a HAKIM™ Programmable Valve Shunt System (Codman & Shurtleff, Inc. Medos S.A. CH 2400 Le Locle, Switzerland), and two patients received a programmable gravitational shunt (proGAV-Shunt system, Aesculap Miethke, Tutlingen, Germany.

### Follow-up and outcome assessment

Follow-up was done in our out-patient clinic at regular time intervals, first at three months. As during the pre-operative examination, the NPH score expressed the combined severity of gait disturbance, urinary incontinence and dementia. If a patient at some time was unable to attend the clinic, he or she was interviewed by phone. The response to shunt surgery was determined after 12 months. We define an increase ≥ 2 scores on our NPH scale as representative of clinical improvement, a change which is generally appreciated by the patients and their families/proxies. Thus, the surgically treated patients were categorized either as Responders (change in NPH score ≥ 2) or Non-responders (change in NPH score < 2), respectively.

### Analysis of pulsatile ICP and pulsatile lumbar CSF pressure

The continuous ICP/CSFP waveforms were stored on a hospital server (sampling rate 100-200 Hz). A method [14] implemented in software (Sensometrics software, dPCom As, Oslo) was used for retrospective analysis of the CSFP and ICP waveforms. In short, the automatic algorithm identifies the cardiac-beat induced single pressure waves within the continuous pressure signal. For each single pressure wave, pulsatility is characterized by the amplitude [pressure difference (dP) from systolic maximum to diastolic minimum; fig 1b]. For each consecutive six-second (6 s) time window (fig 1b, c), the method computes mean wave amplitude (representing the pulsatile pressure), and mean pressure (representing the static pressure). The 6 s parameter values can be plotted against time (fig 1a, d), and average values determined for selected time periods.

The pulsatile ICP was characterized as the average value of ICP wave amplitude over-night from 11 p.m. to 7 a.m. The pulsatile CSFP was determined both as the average of CSFP wave amplitude during the opening phase after lumbar puncture (fig 1d), and also as the average of CSFP wave amplitude during the standardized infusion time of 10 min, i.e. infusion of 15 ml during 10 min at a rate of 1.5 ml/min (fig 1d). We selected a standardized infusion period of 10 min (corresponding to a standardized infusion volume of 15 ml) to be able to compare all the infusion tests.

### Statistics

Statistical analyses were performed in SPSS, version 12.0 (SPSS Inc., Chicago, IL, USA). Differences between groups were determined by one-way ANOVA. Correlations were calculated using bivariate analysis, with determination of Spearman correlations. Significance was accepted at the 0.05 level.

## Results

### Patients

During the period 2002-2007, a total of 214 iNPH patients underwent diagnostic ICP monitoring as part of pre-operative work-up in the Department of neurosurgery. A subgroup of 62 iNPH patients also underwent lumbar infusion testing the day following over-night ICP monitoring (Table 1). Median age of the total material was 72 years; their symptoms had lasted median 2.8 years (Table 1).

**Table 1:** Demographic data of the patient material

	**All patients**	**Shunt Group**	
		
		**Non-Responders**	**Responders**
			
Number	62	10	35
Age (yrs)	72 (37 - 85)	68 (47 - 81)	72 (47 - 81)
Sex (F/M)	31/31	5/5	18/17
Clinical state			
Duration of symptoms (yrs)	2.8 (0.3 - 10)	3 (1 - 10)	3 (1 - 8)
NPH score (15-3)	9 (4 - 14)	11 (6 - 14)	9 (4 - 13)
Radiology			
Evan's index	0.4 (0.3 - 0.5)	0.4 (0.3 - 0.5)	0.4 (0.3 - 0.5)

### Results of diagnostic ICP monitoring and lumbar infusion testing

Diagnostic ICP monitoring caused minor complications in 4 of 62 patients (6.5%), which only included subcutaneous wound infections that were treated with antibiotics without sequels. The lumbar infusion tests caused no complications.

**Figure 2:**
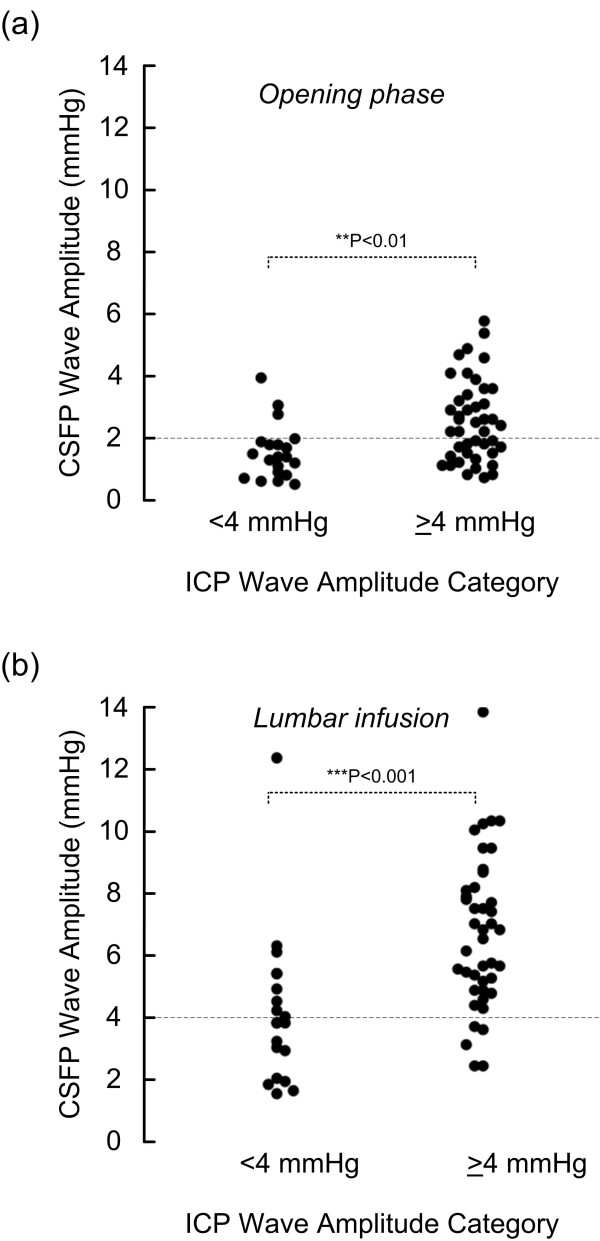
**Scatter plots of CSFP wave amplitude during the opening period and during lumbar infusion for the two categories of over-night pulsatile ICP (mean ICP wave amplitude either < 4 mmHg or ≥ 4 mmHg during the period 11 p.m. to 7 a.m.) a) Shows the pulsatile CSFP (mean CSFP wave amplitude) during the opening phase after lumbar puncture, and (b) during the phase of lumbar infusion (15 ml over 10 min; 1.5 ml/min)**. Dotted lines denote different targets for CSFP wave amplitude during the opening period and during lumbar infusion. Significant differences between groups were tested with one-way ANOVA.

The static ICP was normal in all patients (mean ICP 7.6 ± 4.8 mmHg). During over-night ICP monitoring (11 p.m. to 7 a.m.) the pulsatile ICP was elevated (i.e. mean ICP wave amplitude ≥ 4 mmHg) in 42 of 62 patients (68%; Table 2). The pulsatile CSFP was elevated in the sub-group with over-night pulsatile ICP (fig 2); this was seen during the opening phase after lumbar puncture (fig 2a), but was even more evident during the period of lumbar infusion (fig 2b). Thus, among 42 patients with elevated pulsatile ICP over-night (ICP wave amplitude ≥ 4 mmHg), the pulsatile CSFP was low (CSFP wave amplitude < 2 mmHg) in 17 subjects during the opening phase of lumbar puncture (fig 2a), while pulsatile CSFP was low (CSFP wave amplitude < 4 mmHg) in only 5 subjects during lumbar infusion (fig 2b). The numbers are further detailed in Table 2.

**Table 2:** The CSFP pulse during lumbar puncture *versus *ICP pulse during over-night ICP monitoring

Over-night ICP monitoring*	***Lumbar puncture***			
		
**ICP wave amplitude**	**CSFP wave amplitude during opening phase**		**CSF wave amplitude during lumbar infusion**	
		
	< 2 mmHg	≥ 2 mmHg	< 4 mmHg	≥ 4 mmHg
< 4 mmHg (n = 20)	17	3	12	8
≥ 4 mmHg (n = 42)	17	25	5	37
		
Statistics	Sensitivity = 60; Specificity = 85 PPV = 89; NPV = 50		Sensitivity = 88; Specificity = 60 PPV = 82; NPV = 71	

*Mean ICP wave amplitude from the period 11 p.m. to 7 a.m.

### Clinical response to shunting related to the diagnostic ICP and infusion testing

Forty-five of the 62 patients were shunted, in whom 35 (78%) were shunt responders (Table 1). Major complications to shunt surgery were seen in 18% of patients (chronic subdural haematoma in 7%, shunt infection in 2%, visual failure in 2%, and shunt failure in 7%). Minor complications (headache, abdominal pain, dizziness) were seen in 13%.

Among the 45 patients being shunted, R_out _≥ 12 mmHg/ml/min was seen in 44 patients. Moreover, of these 44 patients, 35 (79.5%) were shunt responders, whereas nine (20.5%) were non-responders. There was a weak, though significant, correlation between R_out _and change in NPH score (i.e. clinical improvement) 12 months after shunting (Spearman correlation 0.31; *P *= 0.04; one way ANOVA; data not shown).

**Figure 3:**
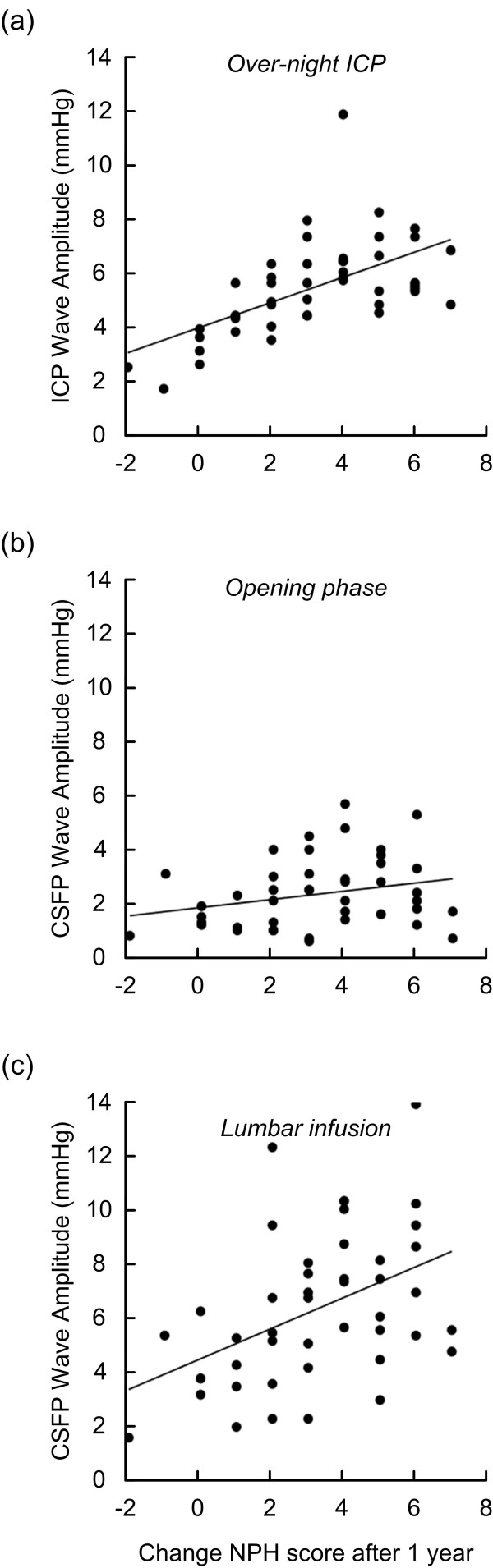
**Graphs showing the correlation between changes in NPH Score one year after shunt surgery and (a) ICP wave amplitude during over-night ICP monitoring, (b) CSFP wave amplitude during the opening phase after lumbar puncture, and (c) CSFP wave amplitude during the phase of lumbar infusion (15 ml over 10 min; 1.5 ml/min)**.

**Figure 4:**
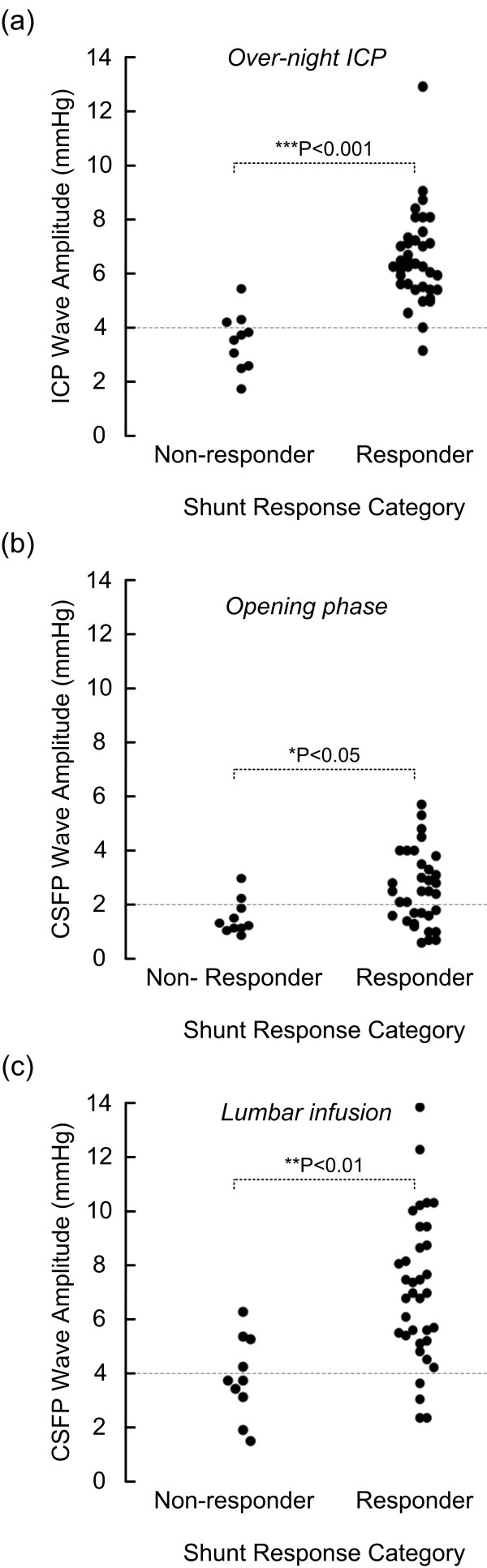
**Scatter plots of mean CSFP wave amplitude for shunt response categories non-responder and responder during (a) over-night ICP monitoring, (b) during the opening phase after lumbar puncture, and (c) during the phase of lumbar infusion**. Dotted lines denote cut off targets for the pulsatile pressure (wave amplitude). The pulse amplitude during the opening phase of lumbar puncture was not as good a predictor for shunt response as the amplitude during overnight monitoring and during lumbar infusion. Significant differences between responders and non-responders are shown (one-way ANOVA).

When correlating the clinical improvement 12 months after shunting (change in NPH score) with the CSFP pulse, we found a highly significant correlation with the ICP pulse amplitude recorded over-night (Spearman correlation 0.58; *P *< 0.001; fig 3a). The clinical improvement after 12 months was not significantly correlated with the CSFP pulse measured during the opening phase after lumbar puncture (Spearman correlation, fig 3b). On the other hand, we found a significant correlation between the change in NPH score after 12 months and the CSFP pulse measured during lumbar infusion (15 ml over 10 min) (Spearman correlation 0.47; *P *= 0.002; fig 3c). These observations are further illustrated in fig 4. The shunt responders and non-responders were best differentiated by the over-night ICP monitoring (fig 4a), but were also differentiated by the CSFP pulse during the opening phase after lumbar puncture (fig 4b) and more so by the pulse during infusion (fig 4c).

The prediction of shunt response from results of ICP monitoring or lumbar infusion testing is presented in Table 3. The data show high positive predictive values (PPV) and negative predictive values (NPV) for ICP pulse amplitude recorded over-night, and also for the CSFP pulse during lumbar infusion.

**Table 3:** Number of shunt responders/non-responders depending on results of lumbar puncture or ICP monitoring

	***Lumbar puncture***				ICP monitoring*	
		
	**CSFP wave amplitude** **during opening phase**		**CSF wave amplitude** **during lumbar infusion**		**ICP wave amplitude**	
			
	< 2 mmHg	≥ 2 mmHg	< 4 mmHg	≥ 4 mmHg	< 4 mmHg	≥ 4 mmHg
Responder (n = 35)	13	22	4	31	1	34
Non-Responder (n = 10)	8	2	6	4	7	3
			
Statistics	Sensitivity = 63; Specificity = 80 PPV = 92; NPV = 38		Sensitivity = 88; Specificity = 60 PPV = 89; NPV = 60		Sensitivity = 97; Specificity = 70 PPV = 92; NPV = 88	

* Mean ICP wave amplitude from the period 11 p.m. to 7 a.m. PPV = Positive predictive value; NPV = Negative predictive value.

## Discussion

The main observation of this study is that the CSFP pulse amplitude determined during lumbar infusion testing showed good correlation with the ICP pulse amplitude recorded over-night, and also with the clinical response to shunting in iNPH. The data support our hypothesis that determining pulsatile CSFP during lumbar infusion can be useful as a screening procedure for selection of iNPH patients for shunting.

### Patients

We have previously reported our entire experience of managing iNPH patients using diagnostic ICP monitoring in the pre-operative work-up during the period 2002-2007 [15]. The present subgroup of 62 iNPH patients managed during the same time period represented the patients that also underwent lumbar infusion testing on day 3. The reason for doing lumbar infusion testing in these patients was that lumbar infusion testing has been done since the 1980's in this department, and thus represented the traditional management. Therefore, we considered how this subgroup compared with our entire cohort of iNPH patients. The present group of 62 patients compared well with our entire cohort during this time period regarding age, sex, symptom duration, severity of symptoms and ventricular size (Table 1) [15]. Moreover, in our entire cohort of 130 shunted iNPH patients, 79% were shunt responders [15], as compared to 78% of 45 shunted iNPH patients in the present study. The present material is therefore representative of our entire experience of managing iNPH. In comparison, McGirth *et al*. [4], using 3-days external lumbar drainage (ELD) and positive finding of A- or B-waves on spinal puncture, found in 132 patients a long term shunt response rate of 75%. Moreover, using gait improvement after 3-days ELD to aid selection for surgery, Marmarou *et al*. [3] reported clinical improvement in 76 (91%) of 84 patients.

### Overnight pulsatile ICP versus pulsatile CSFP during lumbar infusion testing

Based on our experience of diagnostic ICP monitoring in 214 iNPH patients [15], we categorized pulsatile ICP as being elevated when ICP wave amplitude is ≥ 4 mmHg during over-night monitoring. Thus, among 130 shunted iNPH patients, 93% with elevated ICP pulse (i.e. ICP wave amplitude ≥ 4 mmHg during over-night monitoring) were shunt responders whereas only 10% with low ICP pulse (i.e. ICP wave amplitude < 4 mmHg over-night) responded to shunting [15]. In the present group, 68% of patients had elevated ICP pulse.

The present dataset clearly showed that pulsatile CSFP was elevated in those with elevated over-night ICP pulse. This was most evident during the period of lumbar infusion, as compared to the opening phase after lumbar puncture (fig 2; Table 2). Examining simultaneous measurements of lumbar CSFP pulse and intracranial ICP pulse during lumbar infusion testing, we have previously shown in 35,532 CSFP/ICP single wave pairs that the lumbar CSFP wave amplitudes are about 2 mmHg below the simultaneous cranial ICP wave amplitudes [17]. Thus, from this experience, we categorized ICP wave amplitudes ≥ 4 mmHg as indicative of elevated pulsatile ICP [15], and lumbar CSFP wave amplitude ≥ 2 mmHg as indicative of elevated pulsatile CSFP [8,17].

**Figure 5:**
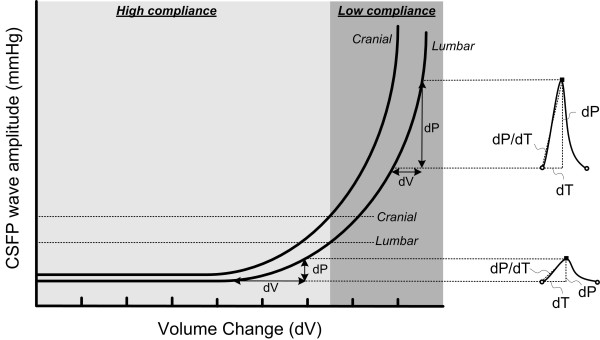
**Assessments of intracranial compliance and lumbar compliance during lumbar infusion**. This diagram illustrates the intracranial and lumbar pressure-volume curves and the relationship to the pulsatility parameters. Under normal physiologic conditions with high intracranial compliance, ICP wave amplitude is correspondingly small. As intracranial compliance decreases (steep part of the pressure-volume curve), the brain behaves increasingly like a linear elastance and so variations in intracranial volume correlate increasingly well with changes in ICP wave amplitude, thus the steepness of the pressure-volume curve accounts for large-amplitude ICP waveforms. The cranial pressure-volume curve is left of the lumbar pressure-volume curve, illustrating that compliance is higher in the lumbar compartment.

One explanation for this discrepancy between the intracranial and intraspinal compartments is the difference in compliance (i.e. pressure-volume reserve capacity) between the compartments. This assumption is further illustrated in fig 5; the pressure-volume curve of the intraspinal compartment is moved to the right as compared to the curve of the intracranial compartment, indicative of higher compliance in the intraspinal than the intracranial compartment. A methodological drawback relates to the fact that the pulsatile CSFP during lumbar infusion testing was measured through the same needle as the infusion. Therefore the pulsatile CSFP during infusion might be slightly increased due to the resistance of the lumbar needle at the infusion rate of 1.5 ml/min. Measurements from a second needle might prevent this effect, and give slightly lower pulsatile CSFP values. However, the impact of this effect is probably minor. Thus, simultaneous measurements of pulsatile ICP and lumbar pulsatile CSFP during lumbar infusion showed that the lumbar CSFP pulse amplitudes were about 2 mmHg below the ICP pulse amplitudes both before and during the infusion [17].

In the present study, we solely focused on the pulsatile pressures. In line with our previous experience [15], over-night static ICP was normal in these patients (hence the term normal pressure hydrocephalus). Others have previously examined differences in the static pressures between intraspinal and intracranial compartments. Several authors have found that the short-lasting opening CSFP measured during the opening phase of lumbar puncture (opening pressure) did not relate well to ICP recorded over-night [17,19,20]. Others found lumbar mean CSFP to agree with the ICP across a large pressure interval [21]. The reason for the discrepancy is that the static pressure depends on the baseline level and zero calibration since the static pressure refers to the difference between the atmospheric pressure and the intra-compartment pressure [22,23]. The pulsatile pressure, characterized by the wave amplitude, on the other hand, refers to the intra-signal difference between the diastolic and systolic pressures (fig 1), thus being independent of the baseline pressure level [22,23]. We have previously shown that the two sensors used for pressure monitoring in this study are equivalent for pulse pressure wave analysis; comparing a total of 218,589 single pressure wave pairs from the intracranial compartment from these two pressure sensors revealed a difference in pulse pressure amplitude of only 0.13 mmHg (95% confidence interval 0.12 -0.13 mmHg) [23].

### Clinical response to shunting related to the diagnostic ICP and infusion testing

Among those 37 patients with elevated ICP pulse overnight (i.e. mean ICP wave amplitude ≥ 4 mmHg; time period 11 p.m. - 7 a.m.), 34 (92%) were shunt responders, while among those eight with low ICP pulse overnight (i.e. mean ICP wave amplitude > 4 mmHg; time period 11 p.m. - 7 a.m.) only one (13%) responded to shunting (Table 3). These numbers compare with our entire series of shunted iNPH patients during this same time period (2002-2007) [15].

During lumbar infusion testing, our standard approach has been to determine resistance to CSF outflow (R_out_). The cut-off value for considering R_out _pathologically elevated varies in the literature. According to our routine, we have considered R_out _≥ 12 mmHg/ml/min as abnormal [6,12]. Therefore, among our 45 shunted iNPH patients, R_out _≥ 12 mmHg/ml/min was seen in all but one patient. Among the resulting 44 patients, 35 (80%) were shunt responders. In general, the role of R_out _in predicting shunt response is still highly disputed [6,8-10,13]. Based on our earlier experience with determination of R_out _[8,11], we have now discontinued using the R_out _for the purpose of selecting iNPH patients for shunting.

In this cohort, we found a highly significant correlation between overnight pulsatile ICP and the degree of clinical improvement seen 12 months after shunting (fig 3a). The correlation between R_out _and clinical improvement was weaker, though it reached significance. These observations compare with our previous experience [8,11,15]. A new observation here was that the CSFP pulse amplitude measured during lumbar infusion (15 ml over 10 min) correlated better with clinical improvement after shunting (fig 3c). This latter observation compares with recently reported observations, focusing on the distribution of the CSFP pulse during lumbar infusion testing [8]. It should be noted that prior to shunting the R_out _and not the CSFP pulse was used to select patients for shunt surgery. As shown in Table 3, clinical response to shunting was best predicted by over-night ICP monitoring, in line with previous observations [15]. The most important observation of this study is that clinical shunt response was also predicted by the CSFP pulse during lumbar infusion (Table 3). Thus, when CSFP wave amplitude was ≥ 4 mmHg during lumbar infusion of 15 ml over 10 min, clinical response to shunting was predicted with a sensitivity of 88 and specificity of 60 (PPV 89; NPV 60).

Taken together, the present results suggest that determining CSFP pulse amplitude during lumbar infusion testing can be useful as a screening procedure for selection of patients to shunt surgery. An advantage of lumbar infusion testing, as compared to over-night ICP monitoring, is that the procedure is a low cost and low threshold approach with few complications that is widely used. Moreover, determining the CSFP pulse was more useful than the R_out _determination.

### Measuring pulsatile CSFP during lumbar infusion versus intracranial compliance

Figure 5 provides a tentative explanation of what is being tested while monitoring pulsatile CSFP during lumbar infusion. We assume that the CSFP pulse provides an indirect measure of the compliance (pressure-volume reserve capacity) of the intraspinal compartment. When the compliance is being reduced, the amplitudes (dP) are increasing [24,25]. The wave amplitude (dP) is the pressure response to the volume change caused by each cardiac contraction, which in the intracranial compartment is about 1 ml [26]. During lumbar infusion the compliance is being artificially reduced (moving to the right on the pressure-volume curve). When compared to the cranial pressure-volume curve, the spinal pressure-volume curve is shifted to the right because compliance in the spinal compartment is higher than in the intracranial compartment [17]. For this reason it is necessary to infuse fluid intrathecally to reach the same amplitude values in the lumbar compartment as in the intracranial compartment. Although the wave amplitudes do not measure compliance directly, they are related to compliance [27] and elevated ICP pulse is associated with reduced intracranial compliance [28]. An important effect of shunting is improved compliance and this is why elevated ICP pulse measured during over-night ICP monitoring and also during lumbar infusion, accurately predicts the shunt response in these patients (Table 3).

## Conclusions

Taken together, determining CSFP pulse amplitude during lumbar infusion in this cohort of iNPH patients was useful for predicting shunt response. The data suggest that the approach can be used for screening of iNPH patients for shunt surgery.

## List of abbreviations

CSFP: Cerebrospinal fluid pressure; dP: Single wave amplitude; ELD: External lumbar drainage; ICP: Intracranial pressure; iNPH: Idiopathic normal pressure hydrocephalus; NPV: Negative predictive value; P_o_: Opening pressure; P_p_: Plateau pressure; PPV: Positive predictive value; R_out_: Resistance to cerebrospinal fluid outflow.

## Competing interests

Dr Eide has a financial interest in the software company (dPCom AS, Oslo) which manufactures software licensed to the Department of Neurosurgery, Oslo University Hospital - Rikshospitalet, and used for analysis of the ICP recordings (Sensometrics Software). Dr. Brean reports no competing financial or non-financial interests.

## Authors' contributions

The authors contributed equally to this work. Both authors have read and approved the final manuscript.
